# Forecasting paediatric malaria admissions on the Kenya Coast using rainfall

**DOI:** 10.3402/gha.v9.29876

**Published:** 2016-02-02

**Authors:** Stella Wanjugu Karuri, Robert W. Snow

**Affiliations:** 1 Spatial Health Metrics Group, Kenya Medical Research Institute-Wellcome Trust Research Programme, Nairobi, Kenya; 2 Nuffield Department of Clinical Medicine, Centre for Tropical Medicine & Global Health, University of Oxford, Oxford, United Kingdom

**Keywords:** malaria, rainfall, Indian Ocean Dipole, seasonality, time-series, auto-regressive models, forecasting

## Abstract

**Background:**

Malaria is a vector-borne disease which, despite recent scaled-up efforts to achieve control in Africa, continues to pose a major threat to child survival. The disease is caused by the protozoan parasite *Plasmodium* and requires mosquitoes and humans for transmission. Rainfall is a major factor in seasonal and secular patterns of malaria transmission along the East African coast.

**Objective:**

The goal of the study was to develop a model to reliably forecast incidences of paediatric malaria admissions to Kilifi District Hospital (KDH).

**Design:**

In this article, we apply several statistical models to look at the temporal association between monthly paediatric malaria hospital admissions, rainfall, and Indian Ocean sea surface temperatures. Trend and seasonally adjusted, marginal and multivariate, time-series models for hospital admissions were applied to a unique data set to examine the role of climate, seasonality, and long-term anomalies in predicting malaria hospital admission rates and whether these might become more or less predictable with increasing vector control.

**Results:**

The proportion of paediatric admissions to KDH that have malaria as a cause of admission can be forecast by a model which depends on the proportion of malaria admissions in the previous 2 months. This model is improved by incorporating either the previous month's Indian Ocean Dipole information or the previous 2 months’ rainfall.

**Conclusions:**

Surveillance data can help build time-series prediction models which can be used to anticipate seasonal variations in clinical burdens of malaria in stable transmission areas and aid the timing of malaria vector control.

## Background

Malaria is a vector-borne disease which is a major threat to child survival across sub-Saharan Africa. The manifold rise in funding for malaria control programmes over the past decade (1, 2) has been associated with a simultaneous decline in child mortality and parasite prevalence (3) which has rekindled hopes of global eradication by the year 2030 (1, 4). It has been assumed that the success in controlling the disease has been, in part, a result of scaled-up distribution of vector control methods such as insecticide-treated bed nets (3, 5) and the replacement of failing first-line therapy drugs chloroquine and suphadoxine-pyrimethamine with artemisinin-based combination (1).

Kilifi, on the Kenyan Coast, is a malaria-endemic region. The most common type of malaria in the region is caused by *Plasmodium falciparum*. The availability and productivity of breeding sites for the *Anopheles* vector is determined by climatic factors such as temperature and rainfall, with rainfall playing a greater role in transmission at the Kenyan coast (6, 7). The annual rainfall at the Kenyan coast occurs in two seasons: the long rains which take place from March to June and the short rains from October to December, with the latter being subject to considerable inter-annual variability (7). Climate variability, such as the El Niño–Southern Oscillation, have been linked to malaria epidemics in the East African region (8–10). The Indian Ocean Dipole (IOD) is a climate mode of coupled ocean and atmosphere variability in the Indian Ocean (11). The intensity of the IOD can be measured by the Dipole Mode Index (DMI) which is defined as the difference between the sea surface temperature anomaly of the western equatorial Indian Ocean (50E–70E and 10S–10N) and that of the south-eastern equatorial Indian Ocean (90E–110E and 10S–0N) (11). The IOD has an influence on the climate of countries that surround the Indian Ocean Basin and has been shown to have an influence on the short rainy season in East Africa (12, 13). A study of malaria in the highlands of Western Kenya found a causal link between the DMI and incidences of malaria (14).

The burden of severe malaria, warranting admission to hospital, among children along the Kenyan coast is acutely seasonal, corresponding to annual periods of rainfall, and varies between years. This variation has been linked to the level of rainfall (7). The intensity of malaria transmission rose during the late 1980s and peaked in the late 1990s before declining again to low levels during the mid-2000s (15). The temporal associations between declining transmission or disease burden with climatic variations, failing efficacy of treatments, or increasing effective intervention are complex and hard to disentangle. In addition to inter-annual seasonality associated with rainfall, malaria transmission has also been shown to exhibit poorly understood long-term cyclic trends (15).

From as far back as the 1920s, predicting the next epidemic has been a major goal in malaria research (16). The timing of vector control or mass drug administration in preparation for epidemics, as well as understanding future trends in disease burden, requires reliable forecasts. Malaria-forecasting models that have been developed vary from dynamic models, stochastic models, or a combination of both (17–20). These models commonly incorporate climatic factors with varying accuracy, and their performance is therefore difficult to assess. The objective of this study was to use malaria and climate information from a regional surveillance in forecasting. In this article, we develop autoregressive integrated moving average models for the proportion of malarial paediatrics malarial admissions to Kilifi District Hospital (KDH) using admission data from 1990 to 2011.

## Material and methods

### Study area and period

Kilifi County, on the Kenyan Coast, covers an area of 12,245 km^2^. KDH is the county referral hospital located in Kilifi town. Data on malaria admissions were obtained from a paediatric ward surveillance system which was initiated in 1989 (5, 21). The data used for this study consist of counts of monthly malaria paediatric admission, for children aged less than 15 years, with a primary admission of malaria confirmed with microscopy. The majority of paediatric malaria admissions (>90%) are aged less than 5 years. The data also contain overall counts of monthly non-malaria paediatric admissions (age 15 years and younger) from January 1990 to December 2011.

The total monthly rainfall (millimetres) from 1974 to 2014 was obtained from a private meteorological station on a sisal plantation in Kilifi, 2 kilometres from KDH. The DMI (11) was obtained from the Commonwealth of Australia Bureau of Meteorology website: www.bom.gov.au/climate/IOD.

For statistical analysis, we used the R-package version 3.1.3. The transformation *y_t_*=arcsin(*p_t_*), where *p_t_* is the monthly proportion of malaria admissions, was adopted for modelling. The purpose of this transformation was variance stabilisation. For scaling and variance stabilisation purposes, rainfall was transformed to *x_t_*=*rain_t_*/1000, where *rain_t_* is monthly rainfall in milliliters. DMI (*z_t_*) was not transformed. The association across and within the three series was studied using the cross-correlation function (CCF), the autocorrelation function (ACF), and the partial autocorrelation function (PACF) (22). Prior to this analysis, the series for *y_t_* was deseasonalised using dummy variables for months in a year and detrended with a cubic spline with nine knots. The series for *x_t_* was deseasonalised using dummy variables for months.

Following the association analysis, three autoregressive (AR) models for *y_t_* were formulated: an AR model for *y_t_* of order 2, an AR model for *y_t_* of order 2 with *x*_*t*−1_ and *x*_*t*−2_ as predictors, and an AR model for *y_t_* of order 2 with *z*_*t*−1_ as a predictor. In all the models, the trend component was represented by either a cubic function or a cubic spline with a knot at January 2003. Time was represented by the number of days from a reference date, assuming all measurements were taken on the first day of the month. The seasonal component of *y_t_* was represented using dummy variables for months. The models were fit via maximum likelihood estimation. The Akaike information criterion (AIC) and the likelihood ratio test (LRT) were used for model comparison. Normal quantile plots of the residuals from each of the three models, as well as plots of their sample ACF and PACF functions, were used to assess model assumptions. Ljung–Box tests on the models’ residuals were used to assess serial auto-correlation.

To assess the predictive ability of the three models, we obtained year-ahead forecasts using all data occurring in and prior to a given reference year. All the data occurring prior to and in the reference year *YY*, with *YY* = 1995*, …*, 2010, were used to refit the model. The fitted model was then used to obtain the next year's forecast. We worked with the assumption that climate information at least 1 month prior was available. We also obtained the 95% confidence intervals for the forecasts on the transformed scale. Exact confidence intervals with 95% confidence level were obtained for the forecasted monthly proportions of malarial admissions. This computation used the median of monthly admission counts from the past data. We compared the forecasted estimates with the true values using the root mean square error (RMSE) function defined as:

$$RMSE=\sqrt{\sum_{t}\frac{{\left(y_{t}-{\hat{y}}_{t}\right)}^{2}}{12}}$$

## Results

The five summary statistics for the data series are shown in Table 1, where *n_t_* represents the total admission in the month *t*, *p_t_* represents the monthly proportion of malarial admissions with the denominator equal to *n_t_*, and *rain_t_* represents monthly rainfall (millimetres). The variable *z_t_* represents the DMI at month *t*. Monthly paediatric admissions range between 117 and 656, and the proportion of these admissions that were diagnosed as malaria ranges between 1 and 78%. The maximum proportion of malaria admissions during the study period occurred in July 1992. December of 1997 was the month with the second highest proportion of malaria admissions in the study period. The maximum monthly rainfall within the study period was 975 mm (October 1997) and was associated with El Niño. During the study period, 37 months had zero rainfall; the most frequent months of zero rainfall were January and February.

**Table 1:** Summary statistics for *n_t_* (Monthly admissions), *p_t_* (proportion of monthly malaria admissions), *rain_t_* (monthly rainfall in mm), and *z_t_* (monthly dipole modal index (DMI)) from January 1990 to December 2011

Variable	minimum	1st quantile	Median	3rd quantile	maximum
*n_t_*	117	247.8	295.5	351.5	656
*p_t_*	0.01	0.14	0.31	0.46	0.78
*rain_t_*	0.00	15.00	51.55	133.57	975.40
*z_t_*	−0.71	0.03	0.19	0.36	1.54

**Fig. 1:**
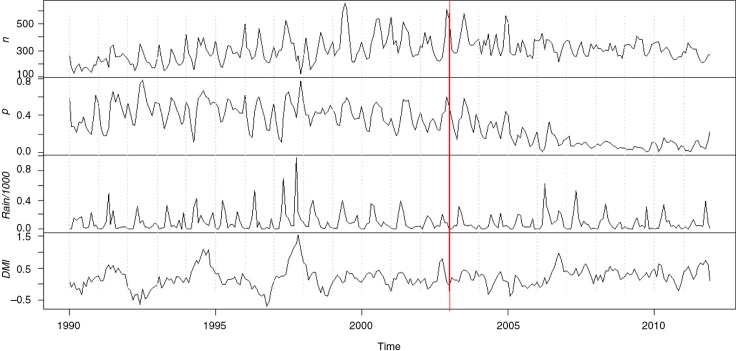
Plots of the series *n_t_*, *p_t_*, *rain_t_*/1000, and *z_t_*; the dashed vertical lines mark the beginning/end of a year. The red line marks January 2003, the beginning of a declining trend in malaria admissions.

Monthly plots of the series are given in Fig. 1. The plots for *p_t_* and *n_t_* exhibit the same rise and fall patterns which indicate malaria as a major cause of admission during the period of observation. The variation in *p_t_* is not constant and decreases with time. The plot also indicates a decreasing non-linear trend in *p_t_* after 2003. With the exception to 1997, the more pronounced within year peaks in rainfall occur within the first half of the year and indicate the long rain season. Less pronounced peaks occur at the end of the year and indicate the short rains. The pronounced peaks in rainfall that occur in 1991, 1997, and 2006–2007 coincided with the positive values of DMI. From the year 2000 till 2011, there is evidence of correlation between DMI and rainfall series, corresponding largely with the short rain season from October to December; this has been documented previously (13).

**Fig. 2:**
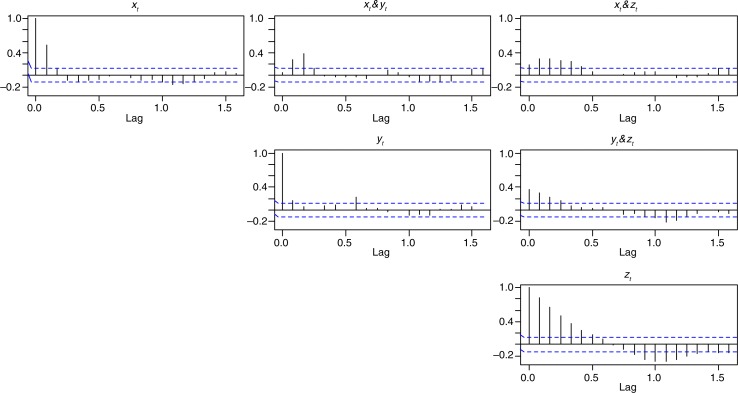
Plots of autocorrelation function (ACF) and cross correlation function (CCF) of the data series.

Figure 2 shows the association between the three series using the CCF and within series association using the ACF. The plots in the diagonal represent the sample ACF of a series. The sample ACF represents the association of the series with itself when the values are shifted by a given number of lags (months). The sample CCF represents the association in two series when one series is shifted forward by a given number of lags (months). In Fig. 2, the series for *y_t_* indicates a strong autocorrelation at a lag of 1 month (0.54, 95% CI: 0.37–0.7066). From the subplot in Row 1 (top row) Column 2 (middle column), the strongest association between proportions of malaria admission and lagged rainfall is positive and occurs at lags of 1 and 2 months. This indicates that high rainfall in the past 2 months is associated with current high proportions of malarial admissions. The CCF plot for *x_t_* and *z_t_* in row 1 (top row) Column 3 (right column) with *x_t_* leading indicates a significant association between rainfall and the first three lags of DMI. Similarly, the CCF plot of *y_t_* and *z_t_* with *y_t_* leading indicates an association between rainfall and the first few lags of DMI. The large ACF values for *z_t_* are caused by the strong autocorrelation in the series at lag 1 which is propagated to later lags. For example, as a consequence of the strong association between *z_t_* and *z*_*t*−1_ and *z*_*t*−1_ and *z*_*t*−2_, the association between *z_t_* and *z*_*t*−2_ (lag 2 autocorrelation) will result in a significant ACF because of the strong association between the intermediate observation *z*_*t*−1_ with both *z_t_* and *z*_*t*−2_.

Three AR models for *y_t_* were fit; these were guided by the analysis of Fig. 2. Model 1 is an AR model for *y_t_* of order 2. Model 2 is an AR model for *y_t_* of order 2 with *z*_*t*−1_ as a predictor. Model 3 is an AR model for *y_t_* of order 2 with *x*_*t*−1_ and *x*_*t*−2_ as predictors. Diagnostics of the fitted models’ residuals did not indicate any departure of the modelling assumptions.

The results of the fit are given in the Supplementary materials. Model 3 resulted in the smallest AIC. The results of a LRT comparing Model 1 with Model 3 indicate that Model 3 is significant (LRT statistics 36.8, *p*-value approximately 0). The coefficients for the dummy variables representing seasonality change significantly in Model 3 compared with those in Model 1. This indicates rainfall seasonality impacts malaria seasonality. The trend parameters in Model 1 remain more or less the same when rainfall information is incorporated, which indicates that rainfall does not explain the changing trend in proportion of malaria admissions.

**Fig. 3:**
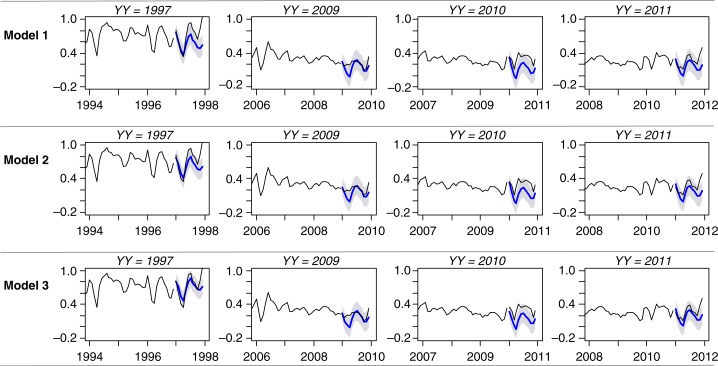
Year-ahead forecasts (on the transformed scale) for years 1997, 2009, and 2010, using previous years’ data. The blue line denotes the forecast, and the black line denotes the observed proportions (on the transformed scale).

**Fig. 4:**
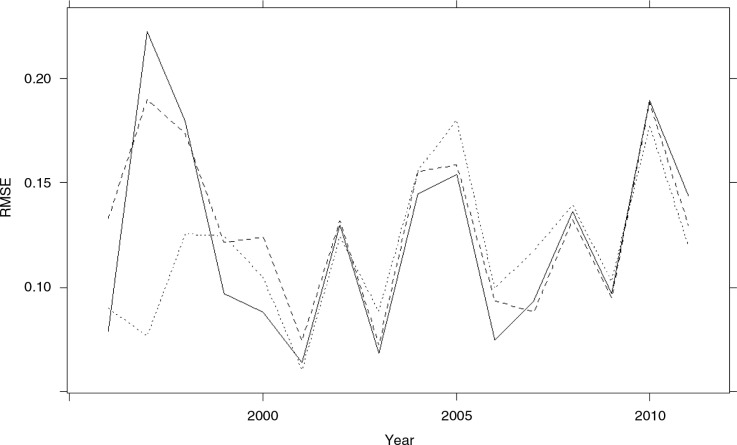
RMSE for 12-month-ahead forecast using previous years’ (1996–2001) data. The x-axis denotes the forecasted year, the solid line represents Model 1, the dashed line represents Model 2, and the dotted line represents Model 3.

To assess the predictive ability of the three models, we obtained 1-year-ahead forecasts using all data occurring in and prior to a given reference year. All the data occurring prior to and in the reference year *YY*, with *YY=*1995*,…*,2010, was used to refit the model. The fitted model was then used to obtain the next year's forecast. We compared the estimates to the true values using the RMSE. Forecasts close to the true value will have a smaller RMSE. Figure 3 gives a year-ahead forecast for the years 1997 and 2009–2011. Predicted proportions of malaria admissions for 1997 are of interest as this year had unusual rainfall because of El Niño. The year 2009 is of interest as this year had the lowest number of malaria admissions, while the year 2010 saw a rise in annual malaria admissions after a decline which started in 2003. The year 2011 is the last year for which data on malaria admissions were available. Model 3 provided considerably better forecasts for the 1997 proportions compared with Model 2. These forecasts also have smaller forecasting errors. All models underestimate *y_t_* in 2010; however, they seem to give better forecasts towards the end of 2009 and the beginning of 2011. The RMSE estimates for the year-ahead forecasts for all models are given in Fig. 4. The three models have comparable prediction accuracy for predictions in 2001–2009, with Model 3 having less accuracy than the other two models in the period 2003–2008. This period coincides to a drought period when rainfall was below average (see Fig. 1). Evidence of the underestimated forecasts for 2010 is given by the large RMSE value for that year. Further studies of the trend in *p_t_* (see Fig. 1) indicates a change in the trend after the year 2003, when there is a much more rapid decrease in *p_t_*; a single cubic trend might not capture this trend.

**Fig. 5:**
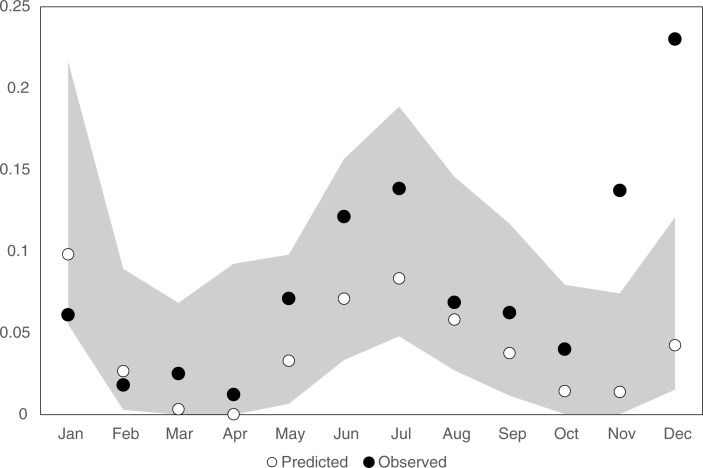
Plot of forecasted proportion of malarial proportions using Model 3. The shaded area gives 95% exact confidence levels.

We model the trend using a cubic spline with a knot at January 2003; this is a segmented cubic trend, with a separate cubic functions prior and post January 2003. This adjustment also captures the rise in malaria admissions that occurred after 2010. The 2011 forecasted proportions after this adjustment for Model 3 are given in Fig. 5, which also gives the 95% confidence level. The plot indicates good predictions and good coverage in the first 10 months.

## Discussion

Linking the incidence of malaria with rainfall is not a new finding; the periodicity of the clinical manifestations of malaria associated with rainfall patterns has been recognised for decades (23, 24). Here, however, we have focussed on the importance of annual seasonal and between-year variations in rainfall, the influence of sea surface temperature, and malaria admissions data alone as predictors of future malaria admissions to hospital. We have developed a time-series model to forecast the monthly rates of malaria admissions to the district hospital in Kilifi based on the data of the previous 2 months. This basic model was improved using ground-station rainfall measurements and IOD indices. As might be expected, the seasonality of malaria burden is linked to seasonal variations in rainfall; with rainfall in the preceding 2 months providing year-ahead prediction with fairly good accuracy such that the forecasts’ RMSE is comparable with the standard error of the modelling errors. Including the DMI reading of the month before hospital admission provided a better fit compared with a model with malaria admission information alone (LRT *p*-value < 0.001). More surprisingly, we show that these models, based on long-term data, can be used to reliably forecast the hospital's malaria burden over the subsequent 12 months (see Figs. 3 and 5).

It is important to note that models based on malaria admission data alone (Model 1) were able to predict subsequent months’ burdens with the same degree of accuracy as Model 2 and Model 3, as shown in Fig. 3. In the absence of any reliable meteorological data proximal to hospitals, this finding suggests that careful monitoring of monthly case burdens will provide valuable information on burdens in subsequent months. However, depending on the needs for more detailed predictions, notably when malaria burdens begin to decline and epidemics might emerge, the inclusion of climate data improves predictive accuracies considerably as shown in Fig. 3. Monthly records of rainfall, carefully collected at a private meteorological station close to the hospital, proved to be a better predictor than the IOD when considered independently. However, our analysis suggests that both predictors result in comparable forecasts when rainfall is not extreme. The fact that the model using dipole information provides comparably even better forecasts raises the possibility of easily available dipole information acting as a surrogate for rainfall where local, reliable meteorological station data are not available.

Most climate prediction models in East Africa have focussed on the margins of stable, endemic transmission in the highland fringes (25–32). However, using time-series prediction models to anticipate seasonal variations in clinical disease burdens has value in other, more stable transmission areas to anticipate surges in hospital caseloads and schedule appropriate community-based actions that might mitigate against exceptional seasonal rises in severe disease.

A limitation encountered in modelling is the adequate representation of the changing trend in malaria transmission. The fit from Model 3 indicates that after accounting for seasonality, rainfall does not impact the trend (on the transformed scale) of the proportion of malaria admissions. Furthermore, the data indicate that the rate of change in the trend of the proportion of paediatric malaria admission, on the transformed scale, is probably caused by extrinsic factors such as increasing drug resistance or the availability of more effective treatment. Cubic splines can accommodate changing trends in malaria transmission. The proposed models are therefore constrained in how far ahead they can forecast before requiring an update. Another limitation in this study is that the modelling did not include patient-level risk indicators such as nutritional status, age, access to treated bed nets, and level of drug resistance. Modelling monthly data and using the assumption that data was collected on the first day of every month helps in ensuring that the numbers in the numerator and denominator of monthly malaria paediatric admissions are sufficient to ensure that the regularity conditions of Models 1–3 are not violated. Using monthly data instead of daily or weekly data and assuming time coincides with the first day of the month has the effect of increasing the modelling error. Consequently, the estimated values of *σ* are underestimates of the true modelling error. Weekly or daily variation in malaria admissions and rainfall can be represented in a Bayesian framework by specifying prior distributions which represent the monthly variation of the data. The applicability of these models are limited to Kilifi and its environs and cannot be extrapolated spatially and we impress similar analyses are required elsewhere to test externality.

## Conclusions

Based on comprehensive surveillance data, we have developed three time-series models to help forecast incidences of paediatric malaria. We have shown that the simplest of the three models, an AR model of order 2, can be used to forecast the malaria hospital burdens over the subsequent 12 months. The more complex models incorporating either past rainfall or dipole information improved this model. We have shown that the trends in these models can be adjusted to accommodate changing trends in malaria prevalence brought on by extrinsic factors such as increasing drug resistance. Though spatially specific to Kilifi, the model structures’ can form the basis of a hierarchical modelling approach encompassing data from various locations.

## Supplementary Material

Forecasting paediatric malaria admissions on the Kenya Coast using rainfall
