# Supplementary material for: Forecasting paediatric malaria admissions on the Kenya Coast using rainfall
Source: Glob Health Action. 2016 Feb 2;9:10.3402/gha.v9.29876. doi: 10.3402/gha.v9.29876 (PMC4740093; doi:10.3402/gha.v9.29876)
Supplement: Forecasting paediatric malaria admissions on the Kenya Coast using rainfall [file GHA-9-29876-s001.pdf]

Supplementary Material

Modeling of Malaria Admissions

We fit three models for the anomaly of y_t, a baseline model comprised of a times series model for y_t, a model incorporating past z_t information to the baseline model and a model incorporating past rainfall information to the baseline model.

Model 1 is the baseline model which is defined as

$$y_t - \mu = \beta_1(y_{t-1} - \mu) + \beta_2(y_{t-2} - \mu) + \sum_{i=1}^{12} \theta_i I_{M_i} + f(x) + \epsilon_t$$

where μ is the sample mean for $y_t, t = 1,...,N$, and $\epsilon_t, t = 1, \ldots, N$ are assumed to be independent Normal variables with zero mean and constant variance σ^2. Model 1 is an autoregressive model for y_t of order 2 adjusting for seasonality and a trend component. Adjustment for seasonality is via dummy variables I_{Mi}, $i = Jan,...,Dec$ with $I_{Mi} = 1$ if the admission was in month i. The parameter θ_i represents the change in y_t from the average attributable to month i. The function $f(s) = \gamma_1 s + \gamma_2 s^2 + \gamma_3 s^3$ is a cubic trend over transformed time (s). The variable s was obtained by standardizing the number of days from a reference point. We made the assumption that measurements were taken on the first day of the month, this assumption was made for ease of computation of date-time objects.

Model 2 used all the terms in Model 1 and included past information of z_t

$$y_t - \mu = \beta_1(y_{t-1} - \mu) + \beta_2(y_{t-2} - \mu) + \sum_{i=1}^{12} \theta_i I_{M_i} + f(x) + z_{t-1} + \epsilon_t,$$

with the same assumptions on the residuals. The rational for including z_{t-1} as a predictor is the significant CCF coefficient between y_t and z_{t-1}. Further evidence arises from the fact that z_t is strongly autocorrelated, the differenced series $z_t - z_{t-1}$ resembles white noise. Model 3 is defined as

$$y_t - \mu = \beta_1(y_{t-1} - \mu) + \beta_2(y_{t-2} - \mu) + \textstyle\sum_{i=1}^{12} \theta_i I_{M_i} + f(x) + x_{t-1} + x_{t-2} + \epsilon_t,$$

and updates Model 1 with past two months rainfall.

Models' estimates

Table of Maximum likelihood estimates for Model 1(M1), Model 2 (M2), Model 3 (M3) Akaike Information Criterion (AIC). Significance codes for p-values: '***' for ≤ 0.001, '**' for ≤ 0.01, '*' for ≤ 0.05; '.' for ≤ 0.1

Coeffice	M1		M2		M3	
y_{t-1}	0.74	*	0.73	*	0.71	***
y_{t-2}	-0.19	*	-0.19	*	-0.12	.
z_{t-1}			0.06	*		
x_{t-1}					0.13	***
x_{t-2}					0.21	***
I_{Jan}	0.16	*	0.15	*	0.07	**
I_{Feb}	0.04		0.03		-0.02	
I_{Mar}	-0.07	*	-0.07	*	-0.09	***
I_{Apr}	-0.10	*	-0.10	*	-0.13	***
I_{May}	0.07	*	0.07	*	-0.01	
I_{Jun}	0.17	*	0.16	*	0.01	
I_{Jul}	0.19	*	0.19	*	0.03	
I_{Aug}	0.14	*	0.13	*	0.03	
I_{Sep}	0.10	*	0.09	*	0.01	
I_{Oct}	0.03		0.01		-0.04	
I_{Nov}	0.04	.	0.03		-0.05	.
I_{Dec}	0.14	*	0.13	*	0.03	
s	-0.24	*	-0.24	*	-0.23	***
s^2	-0.08	*	-0.08	*	-0.07	***
s^3	0.04	*	0.04	*	0.04	**
σ	0.07		0.07		0.07	
AIC	-584		-593		-617	

The coefficients for s, s^2 and $, s^3$jointly characterize the curve. In the equation that defines the cubic trend: $f(s) = \gamma_3 s^3 + \gamma_2 s^2 + \gamma_1 s + d,$ the constant d is determined by the coefficient for the corresponding Month's dummy variable. For example, Aug 2001 has standardized time s=0.098. Using Model 3, the predicted mean for $y_t - \mu$ is $0.14 + \ 0.04 * 0.098^3 - 0.08 * 0.098^2 - 0.23 *$

$0.098 = 0.12$. The rate of change at a point of the response in August 2001 using Model 3 is $f'(0.098) = 3 * 0.04 * 0.098^2 - 2 * 0.08 * 0.098 - 0.23\ =\ -0.25$. The negative value indicating a decreasing trend at this time point. The fact that marginally, the coefficient for $s^3\ is$ significant is evidence that the cubic trend well represents the malaria trend in the data.
